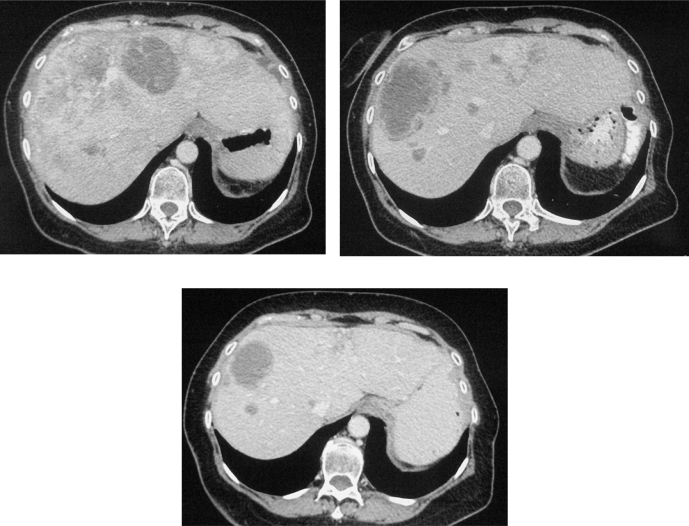# Gemcitabine-induced progressive and sustained tumour response in a patient with multi-drug resistant uterine leiomyosarcoma

**DOI:** 10.3332/ecancer.2008.102

**Published:** 2008-11-24

**Authors:** T De Pas, G Spitaleri, PD Vigna, F Toffalorio, G Curigliano, A Gambino, S Boselli, C Catania, F de Braud

**Affiliations:** 1 Clinical Pharmacology and New Drugs Development Unit, Department of Medicine, European Institute of Oncology, Ripamonti 435, Milan, Italy; 2 Division of Radiology, European Institute of Oncology, Ripamonti 435, Milan, Italy; 3 Ospedali Civili Brescia, Italy

**Keywords:** Gemcitabine, second-line chemotherapy, refractory uterine leiomyosarcoma

## Abstract

**Background:**

despite the fact that the combination of gemcitabine (GCB) and docetaxel shows an increased benefit for disease-free survival and overall survival compared to GCB alone in patients with soft-tissue sarcoma, GCB mono-chemotherapy should be considered as a preferable option with respect to the combination because of its lower toxicity profile and the possibility of it being administered continuously for a long time period.

**Case report:**

we report a clinical case of a woman with advanced high-grade uterine leiomyosarcoma, refractory to ifosfamide, doxorubicin and trabectedin, who experienced a sustained and progressive response to GCB alone.

**Conclusions:**

GCB given as mono-chemotherapy can obtain long-lasting tumour control in patients heavily pre-treated with various chemotherapeutic regimes for uterine LMS and should be considered as a possible option for this subset of patients.

## Introduction

Very few cytotoxic agents have anti-tumour activity in patients with advanced soft-tissue sarcomas (STS) resistant to ifosfamide and anthracyclines. However, Gemcitabine (GCB) has been reported to have a 20% response rate in persistent or recurrent uterine leiomyosarcomas (LMS) [1]. Its preferred use is in combination with docetaxel as it has high activity (53% RR) when used in this regimen in both treated and untreated patients with LMS [2], and due to its higher efficacy in unselected STS compared to GCB alone [3].

We report a clinical case of a woman with advanced high-grade uterine leiomyosarcoma refractory to ifosfamide, doxorubicin and trabectedin, who experienced a sustained and progressive response to GCB alone.

## Case report

A 49-year-old female patient, with no relevant co-morbidities, underwent a hysterectomy with bilateral ovariectomy in 1999 for a high-grade uterine LMS.

On May 2001, because of lung metastases, the patient received six cycles of ifosfamide 9 g/m^2^ and epidoxorubicin 90 mg/m^2^ with further tumour progression.

Between November 2001 and November 2003, she underwent multiple lung wedge resections and two spleno-pancreasectomies for LMS metastases.

Between February and June 2004, because of systemic (lung, mediastinal nodes and chest wall) disease progression, the patient received five cycles of chemotherapy with docetaxel 75 mg/m^2^ and GCB 1000 mg/m^2^. Despite a partial tumour response (RECIST criteria) lasting for six months, this treatment was burdened by high toxicity, such as G4 neutropenia that required granulocyte colony-stimulating factor (G-CSF) support, G3 fatigue and G2 neurotoxicity. Due to disease progression, the patient was started on docetaxel plus GCB in February 2005, leading to disease stabilization. However, the treatment was discontinued due to relevant side effects, thus leading to tumour progression within four months. The patient was sequentially treated with high-dose (12 g/m^2^) ifosfamide, trabectedin and etoposide, but with further progression.

**Figure 1a–c:**
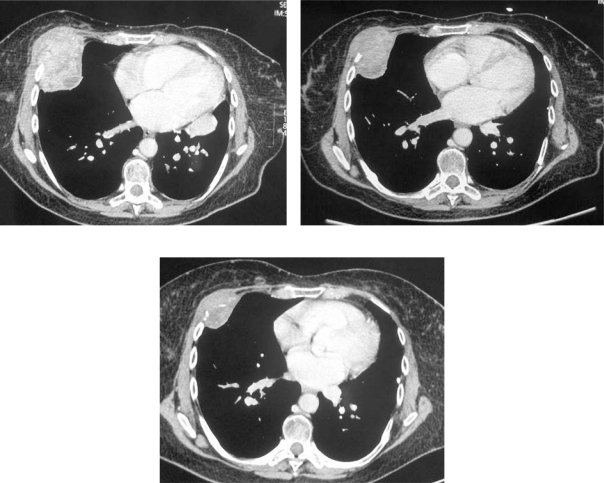


**Figure 2a–c:**
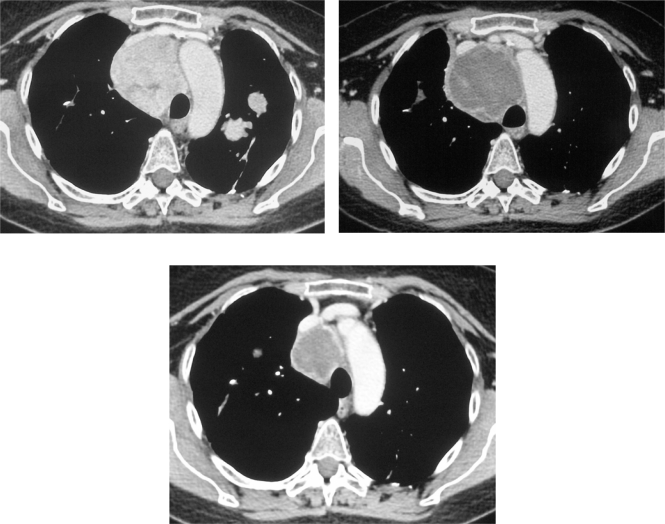


In February 2007, the patient suffered from chest pain (Figure 1a), cough and dyspnea; a bi-weekly 1000 mg/m^2^ GCB administration was started. Within four weeks of the start of treatment the patient obtained complete symptom control, and a subsequent CT scan showed tumour regression. A sustained and progressive tumour response was demonstrated by subsequent CT scans (Figures 1–3), with an overall tumour response lasting more than nine months. At present, the patient is still asymptomatic and the treatment is still ongoing.

## Discussion

Anti-tumour activity has been observed with the GCB and docetaxel combination in patients with advanced sarcoma, especially LMS. Although feasible, this regimen appeared to be burdened by relevant toxicity, with >20% of patients experiencing grade 3 and 4 neutropenia (despite G-CSF as primary neutropenia prophylaxis), thrombocytopenia and fatigue [2–5]. Mono-therapy with GCB has previously demonstrated activity in LMS [1,5].

Despite the fact that docetaxel-GCB combination has shown a more favourable effect on disease-free survival and overall survival than GCB alone in patients with unselected STS [3], the mono-therapy should be a preferable option due to its lower toxicity profile and the possibility for it to be administered continuously for a long-time period.

In our report, GCB attained a sustained and progressive tumour response in a patient with advanced uterine LMS refractory to many chemotherapic agents, including ifosfamide, anthracycline and trabectedin.

While docetaxel-GCB administration had to be discontinued because of toxicity, GCB mono-chemotherapy was very well tolerated and resulted in full symptom control.

The case presented here underlines the value of mono-chemotherapy for the management of advanced LMS. Indeed, gemcitabine when given as a single agent can obtain long-lasting tumour control in heavily pre-treated patients with uterine LMS and should be considered as a possible option for this subset of patients.

## Figures and Tables

**Figure 3a–c:**